# A Case of Miller−Fisher Overlap Syndrome With Positive Anti‐GM4 Antibody and Atypical Symptoms

**DOI:** 10.1002/iid3.70280

**Published:** 2025-10-15

**Authors:** Miao Tang, Runhong Tang, Jian Xu, Zhenyu Yang, Bo Zhang, Yinhua Yan, Jiahui Xie, Qiong Long, Zhi Li, Ewen Tu

**Affiliations:** ^1^ College of Clinical Medicine Hunan University of Chinese Medicine Changsha Hunan China; ^2^ Department of Neurology The Second People's Hospital of Hunan Province (Brain Hospital of Hunan Province) Changsha Hunan China; ^3^ Xiangtan Yuetang District Hetang Neighbourhood Community Health Service Center Xiangtan Hunan China; ^4^ Hunan Society of Traditional Chinese Medicine and Integrated Traditional Chinese and Western Medicine Changsha Hunan China; ^5^ Department of Neurology People's Hospital of Ningxiang City Ningxiang Hunan China

**Keywords:** anti‐GM4 antibody, chlamydial pneumonia, Guillain−Barre syndrome, Miller−Fisher, peripheral neuropathy

## Abstract

**Background:**

Miller−Fisher syndrome (MFS) is a recognized clinical variant of Guillain−Barré syndrome (GBS), characterized by the classic triad of ophthalmoplegia, ataxia, and areflexia. When accompanied by additional symptoms such as bulbar palsy, limb weakness, or lethargy, it is termed MFS overlap syndrome.

**Case Presentation:**

This report describes a male patient diagnosed with MFS overlap syndrome, presenting with ophthalmoplegia, ataxia, bulbar palsy, numbness in both arms, positive GM4 IgG antibodies, a persistent, intractable headache, and a delayed onset of left‐sided peripheral facial palsy. The patient had a preceding suspected case of chlamydial pneumonia before symptom onset, and his condition improved significantly following treatment with intravenous immunoglobulin.

**Conclusion:**

This case suggests that chlamydial pneumonia might predispose individuals to GBS. Patients with MFS/pharyngeal‐cervical‐brachial (PCB) overlap syndrome may exhibit atypical symptoms, including persistent, intractable headaches, and delayed peripheral facial paralysis. Atypical symptoms should not delay the diagnosis and treatment of GBS once other conditions have been adequately excluded. The presence of anti‐GM4 antibodies, often found alongside other anti‐ganglioside antibodies, may serve as a critical immunological factor in MFS/PCB overlap syndrome.

## 1 Introduction

Guillain−Barré syndrome (GBS) is an acute immune‐mediated peripheral neuropathy (PNP), with up to two‐thirds of patients reporting a history of preceding respiratory or gastrointestinal infection [1]. The disease is triggered by cross‐reactivity (molecular mimicry) between a common epitope on peripheral nerves and an immune response to a prior infection or event [2, 3]. Miller−Fisher syndrome (MFS), a recognized clinical variant of GBS, exhibits the classic triad of ophthalmoplegia, ataxia, and areflexia. Atypical manifestations may include headache and delayed facial paralysis [4]. When the classic triad is accompanied by bulbar paralysis, limb weakness, or lethargy, it is classified as MFS overlap syndrome, which encompasses MFS/GBS, Bickerstaff brainstem encephalitis (MFS/BBE), and pharyngeal−cervical−brachial (PCB/MFS) variants [5]. Between 85% and 90% of MFS patients possess anti‐GQ1b antibodies [6]. The specificity of antiganglioside antibodies is associated with the clinical variant of GBS and reflects the distribution of targeted gangliosides in peripheral nerves. GM4 is located in cranial nerves II and VIII, and to a lesser extent in nerves I, V, and VII [4]. Anti‐GM4 antibodies frequently occur alongside other antiganglioside antibodies in immune‐related peripheral neuropathies. This paper presents a case of a patient with MFS/PCB overlap syndrome, who tested positive for anti‐GM4 IgG antibodies and experienced persistent intractable headaches and delayed left facial paralysis, likely triggered by chlamydial pneumonia.

## 2 Case Presentation

**Figure 1:**
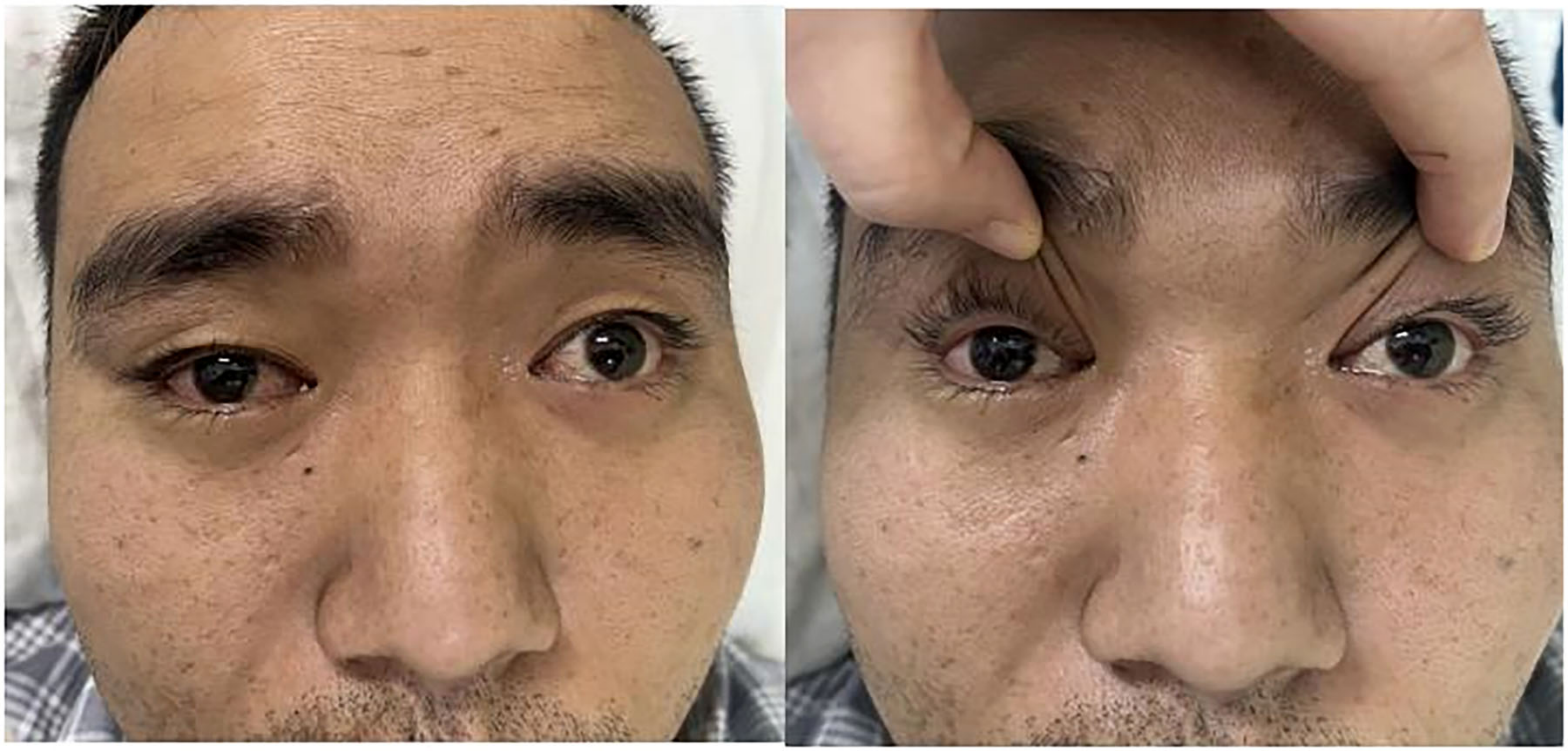
Complete ophthalmoplegia.

A 31‐year‐old man developed fever, cough, and headache following a respiratory tract infection in March 2024. He had a SARS‐CoV‐2 (COVID‐19) Nucleic Acid Test in the local community hospital, and the result was negative. He was treated with Paracetamol, Pseudoepherine Hydrochloride, Dextromethorphan Hydrobromide, and Chlorphenamine Maleate Tablets, and improved, though the headache persisted. Nonsteroidal anti‐inflammatory drugs (NSAIDs) temporarily relieved the headache. After several days of symptomatic improvement, he developed double vision, unsteady walking, slurred speech, drooping of the right upper eyelid, numbness in both upper limbs, and choking when drinking water 3 days later. The symptoms peaked around 3 days later. Previously, his health had been good. Upon admission, the main physical examination findings included: slurred speech, bilateral pupils of equal size and round, about 4 mm in diameter, slightly slow light reflex, complete ophthalmoplegia (Figure 1), right ptosis, limb muscle strength level 5, reduced limb muscle tone, absent tendon reflexes in the limbs, superficial hyperesthesia below the middle of the elbow joint in the upper limbs, and no signs of meningeal irritation or other pathological signs.

Laboratory tests and results: An MRI of the brain with gadolinium was normal. Routine blood tests, liver and kidney function, cardiac enzymes, procalcitonin (PCT), IL‐6, CRP, thyroid‐stimulating hormone, triiodothyronine, thyroxine, tests for Treponema pallidum, hepatitis B and C viruses, human immunodeficiency virus, and viruses such as Influenza A and B, Respiratory syncytial virus (RSV), Adenovirus, and Human Rhinovirus (HRV), were all within normal limits. On the fourth day of illness, which was the seventh day after the onset of fever and cough, we conducted antibody tests for *Mycoplasma pneumoniae* and *Chlamydophila pneumoniae*. *M. pneumoniae* was negative. The *Chlamydia pneumoniae* antibody IgG quantitative was 116.50 AU/mL (the normal range was 0.00−25.00). Coagulation tests showed fibrinogen at 4.77 g/L, within normal range. His acute‐phase serum was analyzed using Enzyme‐Linked Immunospot Assay (ELISPOT) for anti‐ganglioside antibodies, including sulfatides, GM1, GM2, GM3, GM4, GD1a, GD1b, GD2, GD3, GT1a, GT1b, and GQ1b, detecting only anti‐GM4 IgG antibodies; all other antigens tested negative. On the fourth day of illness, a lumbar puncture was performed. The cerebrospinal fluid pressure was 170 mmH_2_O, with normal routine and biochemical results (Table 1). Blood electrolytes and sugar levels were also normal during this period. Tests for tuberculosis, fungal infections, and general bacteria in the cerebrospinal fluid were negative. Both blood and cerebrospinal fluid tests for MOG, GFAP, and AQP4 were negative. Cerebrospinal fluid next‐generation sequencing (NGS) was negative. Peripheral nerve electromyography showed no abnormalities on the seventh day after symptom onset (Table 2).

**Table 1:** Cerebrospinal fluid examinations.

Cerebrospinal fluid	Detection value	Reference range
Biochemical examination		
Total protein (mg/L)	360.4	150.0−450.0
Chlorine (mmol/L)	124.7	119.0−129.0
Glucose (mmol/L)	3.39	2.50−4.50
Routine		
Total number of cells (×10*6)	3	0−8
White blood cell count (×10*6)	0	0−8
Pathogen examinations		
Mycobacterium tuberculosis DNA assay	Not detected	
Ink staining	No cryptococcus	

**Table 2:** Results of motor and sensory nerve conduction studies.

		Latency (ms)		Amplitude (mV)		Conduction velocity (m/s)	
Nerve conduction study	Stimulation point	L	R	L	R	L	R
*Motor NCS*							
Tibial nerve	Ankle	5.05	3.85	14.22	14.29		
	Popliteal fossa	11.05	11.5	12.29	11.63	63.3	51.0
Common peroneal nerve	Ankle	3.6	4.45	9.40	7.05	51.1	47.8
	Head of fibula	10.25	11.15	8.54	7.27		
	Lateral malleolus		5.25		1.32		
Median nerve	Wrist	3.55	3.65	11.07	10.28		
	Elbow	7.7	7.45	10.71	9.94	56.6	61.8
Ulnar nerve	Wrist	2.35	2.5	11.00	11.29		
	Proximal to the elbow	8.2	8.25	10.84	11.24	48.8	50.0
	Distal to the elbow	6.15	6.25	12.27	11.22	55.3	60.0
*Sensitive NCS*							
Superficial peroneal nerve	Lateral lower leg	2.14	1.6	15.80	12.90	51.4	56.3
Sural nerve	Posterior lower leg	2.76	2.66	24.70	19.70		41.4
Median nerve	Wrist	2.62	2.76	23.10	23.10	59.2	56.2
Ulnar nerve	Wrist	2.56	2.44	14.90	12.40	46.9	53.3

Abbreviations: L = left, R = right.

Diagnosis: The patient is a middle‐aged male who had caught a cold before the onset of the disease. His symptoms include dizziness, slurred speech, unsteady walking, coughing when drinking water, numbness of both upper limbs, double vision, and headache. The peak of the disease occurs in about 3 days. Combined with the patient's symptoms and physical examination, it is located in the oculomotor nerve, abducens nerve, trochlear nerve, glossopharyngeal vagus nerve or nucleus ambiguus, cerebellum or its afferent and efferent fibers, and intracranial pain‐sensitive structures. The possibility of etiology is inflammation and immunity. After admission, we performed a plain‐enhanced MRI scan of the head and found no structural abnormalities, which does not support brainstem stroke, brain tumor, brainstem compression, or Wernicke's encephalopathy. The patient had fever and headache before the onset of the disease, we further enforce the lumbar puncture examination of routine, biochemistry, cerebrospinal fluid tuberculosis smear, ink staining, general bacterial smear, cerebrospinal fluid NGS, and blood and cerebrospinal fluid demyelination. No abnormalities were found, which did not support the cerebrospinal membrane inflammation, neuromyelitis optica, or MOG antibody‐related diseases. There were no abnormalities in the electromyography of the patient's peripheral nerves, which did not support anterior horn cell lesions such as poliomyelitis, myopathy, or CIDP. The patient's symptoms had no fluctuation, no morning lightness or evening weight, and combined with numbness of both upper limbs, which did not support myasthenia gravis. The patient was previously in good health and had no history of food, drug, or environmental poisoning before the onset of the disease, which does not support toxic metabolic diseases. In summary, the patient presents with MFS (ophthalmoplegia, ataxia, loss of tendon reflexes, right ptosis) and PCB (slurred speech, regurgitation while drinking, numbness in both upper limbs), leading to a diagnosis of MFS/PCB overlap syndrome.

Treatment and prognosis: The patient received intravenous immune globulin (IVIG) at a dose of 2.0 g/kg, administered over 5 days. Concurrently, he underwent neurotrophic and neurological rehabilitation. Following treatment, the patient could walk independently, and his speech almost normalized. The right ptosis improved, and eye movement remained stable. However, left facial nerve paralysis developed during recovery. Six months after discharge, he returned for a follow‐up examination, at which point the left facial nerve paralysis had completely resolved.

## 3 Discussion

GBS is a heterogeneous syndrome. Wakerley et al. [7] classified GBS into classic GBS (symmetrical flaccid limb weakness), focal GBS (pharyngeal‐cervicobrachial weakness, acute oropharyngeal weakness, paraplegic weakness, and bilateral facial paralysis with paresthesia), classic MFS syndrome, and MFS subtypes. These subtypes include CNS subtype BBE and incomplete MFS variants such as acute ophthalmoplegia, acute ataxic neuropathy, acute ptosis, acute mydriasis, and acute ataxic somnolence, based on the pattern of limb and cranial nerve involvement and the presence of consciousness or vertebral tract changes. Simple MFS presents only the clinical triad of ophthalmoplegia, ataxia, and areflexia throughout the disease course [7]. If symptoms like bulbar paralysis, limb weakness, or lethargy persist, they are classified as MFS overlap syndrome, including MFS/PCB, MFS/GBS, and MFS/BBE [5], typically manifesting other symptoms within 7 days of onset [8]. In our case, the patient exhibited MFS (ophthalmoplegia, right ptosis, ataxia, and areflexia) and PCB (slurred speech, regurgitation while drinking water, and numbness in both upper limbs), supporting a diagnosis of MFS/PCB overlap syndrome.

An analysis of atypical clinical manifestations in 38 MFS patients found that 29% experienced symptoms beyond the classic triad, such as headache, delayed facial palsy, divergence insufficiency, dysgeusia, optic neuropathy, and dysuria [9, 10]. Headaches may precede, coincide with, or follow the onset of ophthalmoplegia and can vary in location and nature, including periorbital, temporal, or generalized brain areas, with descriptions ranging from dull to sharp pain. Most patients report that oral NSAIDs are ineffective, yet these headaches typically resolve within 2 weeks from onset [9]. The pathogenesis remains uncertain, but it is hypothesized that activation of the trigeminovascular pain pathway by anti‐ganglioside antibodies causes demyelination of cervical and intracranial sensory nerves, leading to headaches [11]. Although the GQ1b IgG antibody, closely associated with MFS, is not prevalent in the trigeminal nerve and cervical nerve dorsal root ganglia, other gangliosides enriched in these areas, such as GD3 and GD1b, have been implicated [4], explaining the rarity of pain in MFS. In our case, the patient's headache began before the ophthalmoplegia and was resistant to NSAIDs, persisting until several days after the illness peaked. No evidence of increased intracranial pressure or meningeal or intracranial lesions was found on lumbar puncture, suggesting that GM4 IgG antibodies might also trigger activation of the trigeminovascular pain pathway. Approximately 8% of MFS patients develop delayed facial paralysis as other neurological signs begin to improve [9], typically appearing around 12 days after initial symptoms. The mechanism of delayed facial paralysis remains unclear, but it is thought to be a reversible descending paralysis involving cranial nerves, indicative of a favorable prognosis [12]. Our patient developed left facial paralysis as symptoms improved; however, this did not worsen other symptoms or hinder recovery. Atypical clinical manifestations can complicate MFS diagnosis but broaden its clinical spectrum and may relate to the presence of other anti‐ganglioside antibodies, in addition to anti‐GQ1b, in MFS patients.

The International GBS Outcome Study indicated that 76% of patients experienced precipitating events within the 4 weeks preceding GBS [13], including upper respiratory tract infections (35%) and gastroenteritis (27%). *Campylobacter jejuni* gastroenteritis is the most prevalent prodromal event for GBS, occurring in about 25% of cases. *C. pneumoniae* is a leading cause of community‐acquired pneumonia. In a prospective study involving 95 children with GBS, *C. pneumoniae* was detected in 8% of the cohort [14]. Prior research has linked GBS with GM1 IgG antibodies [15], combined central and peripheral nervous system disease with MOG and sulfatide antibodies (MOGAD + AMSAN) [16] to *M. pneumoniae* infection. Reports also exist of antibody‐negative BBE and GBS overlap syndrome [17]. The patient presented with fever, cough and sputum production, and we initially screened the infection of common pathogens. Influenza A and B, RSV, Adenovirus, HRV, and SARS‐CoV‐2 were negative. The atypical pathogen *M. pneumoniae* is negative, while fungal and Toxoplasma infections are more common in immunocompromised patients. Only *C. pneumoniae* antibody increased, suggesting that *C. pneumoniae* may be the cause of GBS spectrum disease. No prior studies have reported an overlap syndrome involving *C. pneumoniae* with MFS/PCB/GBS. The association between *C. pneumoniae* and the GBS spectrum, as well as its pathogenesis, requires further investigation.

Antiglycolipid antibodies are associated with various acute and chronic autoimmune‐mediated peripheral neuropathies and are considered potentially pathogenic in these conditions. In *C. jejuni*‐associated GBS, strong evidence supports molecular mimicry between peripheral nerves and microbial antigens as a contributing factor to GBS development [18]. GBS subtypes are typically associated with specific anti‐ganglioside antibodies. For instance, AMAN patients often possess IgG antibodies against GM1, GM1b, GD1a, and GalNAc‐GD1a [19], while MFS patients frequently have anti‐GQ1b IgG antibodies [20]. Pharyngocervicobrachial weakness‐type GBS is usually linked to anti‐GT1a IgG antibodies. Studies have shown that MFS/PCB overlap is primarily related to anti‐GQ1b antibodies [21], and partly to anti‐GD1b, anti‐GT1b, and anti‐GM1 IgG antibodies [22, 23]. Additionally, some patients with MFS/GBS overlap syndrome are antibody‐negative [24]. Anti‐GM4 antibodies were first identified in patients with unclassified PNP in 2007, and their coexistence with other anti‐ganglioside antibodies has been observed in GBS, MFS, and multifocal motor neuropathy (MMN) [25]. A study examining ganglioside IgG and IgM antibodies in 290 patients with PNP [26] found GM4 IgG and GM1 IgG co‐occurred in one GBS patient and two patients with possible immune‐mediated neuropathy. High titers of anti‐GM4 IgG antibodies have also been observed in patients with MFS/GBS overlap syndrome [27]. GM4, a less‐frequently discussed monosialic ganglioside, is found in high amounts in myelin and to a lesser extent in astrocytes [28]. Anti‐GM4 antibodies have attracted little attention, perhaps because anti‐GM4 antibodies mostly coexist with other antiganglioside antibodies (AGAbs) in PNP [27]. Biochemical analysis of ganglioside fractions from cranial nerves and spinal ganglionic, preganglionic, and posterior roots indicated that GM4 is present in cranial nerves II and VIII and to a lesser extent in cranial nerves I, V, and VII [4]. The patient in this case was diagnosed with MFS/PCB overlap syndrome and had high titers of monospecific anti‐GM4 IgG antibody in serum without other antiganglioside antibodies that have been reported in previous studies on overlapping MFS/GBS. Although the patient's positive anti‐GM4 IgG antibody does not fully explain every clinical feature observed, this does not relegate it to a mere bystander. We believe the anti‐GM4 antibody may be a critical immunological factor in MFS/GBS overlap syndrome. Larger mechanistic studies focused on the pathogenesis of GM4 in GBS are therefore warranted to expand the recognized spectrum of this disorder.

## 4 Conclusion

The findings of this study indicate that chlamydial pneumonia may serve as a predisposing event for GBS. Patients with MFS/PCB overlap syndrome may exhibit atypical symptoms, including persistent, intractable headaches, and delayed peripheral facial paralysis, in addition to classic clinical symptoms. Diagnosis and treatment of GBS should not be delayed once other diseases have been excluded, despite the presence of atypical symptoms. Anti‐GM4 antibodies frequently occur alongside other anti‐ganglioside antibodies and it may serve as a critical immunological factor in MFS/PCB overlap syndrome.

## Author Contributions

All authors contributed to the study conception and design. All authors read and approved the final manuscript. J.X. and Z.Y.Y. contributed to resources and data curation. B.Z. and Y.H.Y. contributed to methodology. J.H.X. and Q.L. contributed to formal analysis. M.T. and R.H.T. contributed to the validation and writing of the original draft. Z.L. and E.W.T. contributed to funding acquisition, project administration, writing, review and editing.

## Ethics Statement

Our institution does not require ethical approval for reporting individual cases or case series.

## Consent

Written informed consent was obtained from the patient for publication of this case report and accompanying images.

## Conflicts of Interest

The authors declare no conflicts of interest.

## Data Availability

All data are available from the corresponding author upon reasonable request.
